# Surgical Perspectives of Open vs. Laparoscopic Approaches to Lateral Pancreaticojejunostomy: A Comprehensive Review

**DOI:** 10.7759/cureus.51769

**Published:** 2024-01-06

**Authors:** Krushank Nayak, Raju K Shinde, Rajesh G Gattani, Tosha Thakor

**Affiliations:** 1 General Surgery, Jawaharlal Nehru Medical College, Datta Meghe Institute of Higher Education and Research, Wardha, IND; 2 Pathology, American International Institute of Medical Sciences, Udaipur, IND

**Keywords:** surgical outcomes, 3d visualization, surgical dexterity, robotic-assisted surgery, minimally invasive surgery, pancreaticojejunostomy

## Abstract

Pancreaticojejunostomy, a critical step in pancreatic surgery, has significantly evolved surgical approaches, including open, laparoscopic, and robotic techniques. This comprehensive review explores open surgery's historical success, advantages, and disadvantages, emphasizing surgeons' accrued experience and familiarity with this approach. However, heightened morbidity and prolonged recovery associated with open pancreaticojejunostomy underscore the need for a nuanced evaluation of alternatives. The advent of robotic-assisted surgery introduces a paradigm shift in pancreatic procedures. Enhanced dexterity, facilitated by wristed instruments, allows intricate suturing and precise tissue manipulation crucial in pancreatic surgery. Three-dimensional visualization augments surgeon perception, improving spatial orientation and anastomotic alignment. Moreover, the potential for a reduced learning curve may enhance accessibility, especially for surgeons transitioning from open techniques. Emerging technologies, including advanced imaging modalities and artificial intelligence, present promising avenues for refining both open and minimally invasive approaches. The ongoing pursuit of optimal outcomes mandates a judicious consideration of surgical techniques, incorporating technological advancements to navigate challenges and enhance patient care in pancreaticojejunostomy.

## Introduction and background

Pancreaticojejunostomy stands as a critical surgical procedure in the field of pancreatic surgery, representing a key component in the management of various pancreatic disorders. This intricate procedure involves the anastomosis of the pancreas to the jejunum, often performed in the context of pancreaticoduodenectomy or other pancreatic resections. The roots of pancreaticojejunostomy trace back through the annals of surgical history, evolving from rudimentary techniques to contemporary approaches that reflect the ongoing progress in surgical methodologies [1].

Pancreaticojejunostomy, commonly called the connection between the pancreatic remnant and the jejunum, is a surgical technique that plays a pivotal role in the reconstruction phase following pancreatic resections. Initially introduced as part of the Whipple procedure for treating pancreatic tumors, this procedure has undergone substantial refinement over time, adapting to advancements in surgical techniques and technology. The complexity of the pancreaticojejunostomy lies in the delicate nature of the pancreatic tissue and the necessity to establish a secure and functional connection with the gastrointestinal tract [2].

The significance of pancreaticojejunostomy transcends its technical intricacies, as it directly influences postoperative outcomes and patient well-being. Successful pancreaticojejunostomy is imperative for preventing postoperative complications such as pancreatic fistulas, a significant concern in pancreatic surgery. Moreover, this procedure bears substantial importance in preserving the endocrine and exocrine functions of the pancreas, which is crucial for the patient's overall health and metabolic equilibrium [3].

This comprehensive review aims to delve into the comparative perspectives of the open and laparoscopic approaches to lateral pancreaticojejunostomy. As surgical techniques evolve, clinicians face the dilemma of choosing the most optimal approach for their patients. This review aims to provide a detailed analysis of both open and laparoscopic techniques' historical context, procedural nuances, advantages, disadvantages, and outcomes. By synthesizing existing literature and exploring recent advances, this review offers valuable insights into the current state of pancreaticojejunostomy, aiding surgeons in making informed decisions regarding the selection of surgical approaches. Through critically examining the available evidence, we aim to contribute to the ongoing discourse surrounding this crucial aspect of pancreatic surgery, paving the way for enhanced patient care and improved surgical outcomes.

## Review

Open surgical approach

Procedure Details

Incision and exposure: A carefully planned midline abdominal incision characterizes the initial phase of the open surgical approach to pancreaticojejunostomy. This incision, although typically along the midline, may be subject to variations based on the unique anatomy of the patient and the specific pathology of the pancreatic disorder under consideration. The incision choice aims to optimize exposure while minimizing trauma to surrounding tissues [4]. The midline incision is strategically selected to provide optimal access to the pancreas, offering a direct and unobstructed view of the surgical field. This exposure is instrumental in facilitating a comprehensive examination and manipulation of the pancreatic remnant. Given the intricate nature of pancreaticojejunostomy, this extensive exposure is indispensable for the surgeon to navigate the complex anatomy, identify critical structures, and execute subsequent steps with precision [5]. The careful management of the midline incision allows for controlled exploration of the pancreas, minimizing the risk of inadvertent injury to adjacent organs. This deliberate approach sets the foundation for the procedure's success, as the quality of exposure directly influences the surgeon's ability to perform a meticulous anastomosis between the pancreatic duct and the jejunum [6].

Technique for pancreaticojejunostomy: Following the midline incision and exposure, the technique for open pancreaticojejunostomy involves a series of critical steps designed to achieve a secure and functional anastomosis. Once the pancreas is visible, the surgeon meticulously identifies the pancreatic duct, a delicate and vital structure. Careful dissection ensues to isolate the pancreatic duct while preserving its integrity [6]. Subsequently, a segment of the jejunum is selected as the site for anastomosis. This decision is guided by proximity to the pancreas and the feasibility of creating a viable connection. The surgeon then proceeds to establish a precise and secure connection between the pancreatic duct and the chosen segment of the jejunum. This connection is typically accomplished using sutures, applied with high skill and precision [7]. The sutures are crucial in achieving a watertight seal, preventing leakage, and minimizing the risk of postoperative complications, particularly pancreatic fistulas. The procedure's success is contingent upon the surgeon's meticulous attention to detail during this phase, as any imperfections in the anastomosis may have significant implications for patient outcomes [8].

Advantages

Historical success: The open surgical approach to pancreaticojejunostomy is a testament to a robust and successful historical legacy. Over several decades, surgeons have consistently relied on the open technique to address a spectrum of pancreatic disorders, refining and advancing surgical methodologies. A wealth of accumulated knowledge underpins the historical success of open pancreatic surgery, garnered through the extensive collective experience of surgeons worldwide [1]. Throughout the evolution of pancreaticojejunostomy techniques, surgeons have encountered and overcome many challenges, ranging from anatomical variations to complexities in tissue manipulation. This historical context has served as a crucible for innovation, driving the development of nuanced surgical strategies that have stood the test of time. As a result, the open approach has become deeply ingrained in the surgical landscape, with a track record of positive patient outcomes and a legacy of continuous improvement [9]. The historical success of the open approach is not merely a narrative of triumph but a repository of invaluable insights into the intricacies of pancreatic anatomy and the dynamics of surgical intervention. This wealth of historical experience provides contemporary surgeons with a foundation they can build, fostering a sense of confidence and competence as they navigate the challenges inherent in pancreaticojejunostomy [10].

Surgeon familiarity and experience: Surgeons who choose the open approach to pancreaticojejunostomy often do so after years of dedicated training and extensive practice. Becoming proficient in open pancreatic surgery involves a significant investment of time and effort, developing a high level of familiarity and expertise. Through this journey, surgeons cultivate a deep understanding of the nuances associated with the open technique, allowing them to navigate the procedure efficiently from experience [11]. The familiarity that surgeons gain with the open approach is a product of exposure to diverse patient cases and the ability to adapt to variations in anatomy. This adaptability is crucial in pancreatic surgery, where each case may present unique challenges. Surgeons who have honed their skills through open procedures are adept at making precise intraoperative decisions, even in the face of unexpected complexities [12]. The preference for the open approach among experienced surgeons is not solely based on tradition but is rooted in a genuine confidence in the technique's efficacy. The reliability of the open method, coupled with the surgeon's familiarity and adaptability, contributes to a sense of assurance in decision-making during the critical stages of pancreaticojejunostomy [13].

Disadvantages

Increased morbidity: The historical success of the open surgical approach to pancreaticojejunostomy is juxtaposed with notable drawback-increased morbidity when compared to minimally invasive techniques. This elevated morbidity is primarily attributed to the larger incision and more extensive tissue dissection inherent in the open approach. While providing critical exposure for the surgeon, the incision introduces a set of challenges that can contribute to postoperative complications [14]. The larger incision increases the susceptibility to wound infections, posing a potential threat to the patient's overall recovery. The increased surface area of the incision provides a larger target for bacterial colonization, necessitating vigilant postoperative care to mitigate the risk of infectious complications. Additionally, the extended tissue dissection in open surgery can lead to delayed gastric emptying, a standard postoperative issue that may further contribute to patient discomfort and recovery challenges [15].

Pancreatic fistulas, a significant concern in pancreatic surgery, are also more frequently observed in the open approach. The complexity of the pancreaticojejunostomy and the inherent vulnerability of the pancreatic tissue increases the risk of complications, with pancreatic fistulas representing a potential consequence of the more invasive nature of the procedure [16]. The association between increased morbidity and the open approach highlights the ongoing quest for surgical strategies that balance the need for exposure with the imperative to minimize postoperative complications. While the open technique has demonstrated historical success, surgeons must weigh its benefits against the potential drawbacks, especially when considering the evolving landscape of minimally invasive alternatives [17].

Prolonged recovery: The recovery period following open pancreaticojejunostomy is characterized by its often extended duration compared to laparoscopic approaches. The larger incision and more incredible tissue trauma inherent in open surgery contribute significantly to this prolonged recovery, impacting various facets of the patient's postoperative experience [18]. While essential for optimal exposure, the larger incision is associated with heightened pain and discomfort. This, coupled with the extended tissue dissection, necessitates a more cautious and deliberate recovery process. The prolonged hospital stays, often required for careful postoperative monitoring and management of potential complications, represent an additional aspect of the extended recovery period [19]. The delayed return to normal activities for patients undergoing open pancreaticojejunostomy poses challenges to their overall quality of life. The physical limitations imposed by the prolonged recovery may impact daily functioning and temporarily reduce the patient's ability to engage in routine tasks. Moreover, the extended recovery period strains healthcare resources, including increased hospitalization costs and a greater demand for postoperative care services [20]. Recognizing and addressing the prolonged recovery associated with open surgery is pivotal in enhancing patient outcomes and satisfaction. As advances in surgical techniques continue, minimizing the recovery burden on patients remains a critical goal, prompting a nuanced evaluation of the trade-offs between the benefits of the open approach and the potential impact on the postoperative recovery trajectory [21].

Laparoscopic surgical approach

Procedure Details

Trocar placement and exposure: The laparoscopic approach to pancreaticojejunostomy involves using small incisions and trocars to access the abdominal cavity. Trocar placement is strategically chosen to optimize the pancreas's and surrounding structures' exposure. Three to four trocars are typically inserted, including a camera port for visualization and working ports for instruments. Once access is established, the pneumoperitoneum facilitates a working space and enables laparoscopic manipulation [22].

Technique for laparoscopic pancreaticojejunostomy: The laparoscopic technique for pancreaticojejunostomy requires high skill and precision. After trocar placement, the surgeon employs laparoscopic instruments to identify and dissect the pancreatic duct and the jejunum. The anastomosis is then performed using a combination of laparoscopic suturing and stapling techniques. Advanced energy devices and hemostatic agents aid in achieving a secure and leak-proof connection between the pancreatic remnant and the jejunum. Throughout the procedure, the surgeon relies on laparoscopic imaging for visualization, ensuring accurate placement of sutures and optimal anastomotic alignment [23].

Advantages

Minimally invasive nature: One of the primary advantages of the laparoscopic approach to pancreaticojejunostomy is its minimally invasive nature. The use of small incisions reduces tissue trauma, postoperative pain, and the risk of wound complications. Patients undergoing laparoscopic procedures often experience a faster recovery and improved cosmetic outcomes compared to traditional open surgery [24].

Reduced hospital stays: The minimally invasive nature of laparoscopic pancreaticojejunostomy contributes to shorter hospital stays for patients. The reduced postoperative pain and quicker recovery allow for an earlier return to normal activities. This advantage benefits the patient's overall experience and contributes to more efficient healthcare resource utilization [25].

Disadvantages

Learning curve: The laparoscopic approach to pancreaticojejunostomy requires a significant learning curve for surgeons. Mastering the skills necessary for laparoscopic suturing, tissue manipulation, and three-dimensional (3D) visualization can be challenging. Surgeons transitioning from open to laparoscopic techniques must undergo specialized training to ensure proficiency and minimize the risk of intraoperative complications [26].

Technical challenges: Laparoscopic pancreaticojejunostomy poses technical challenges related to the limited range of motion and reduced tactile feedback compared to open surgery. Precise instrument control and spatial orientation are crucial for successful outcomes, and the laparoscopic surgeon must navigate these challenges adeptly. Additionally, managing bleeding and achieving hemostasis can be more intricate in the laparoscopic setting [27].

Comparative analysis

Outcomes

The search results show a comparative analysis of open and laparoscopic approaches to pancreaticojejunostomy, focusing on postoperative complications and long-term survival rates. Laparoscopic pancreaticoduodenectomy (LPD) has advantages such as less intraoperative blood loss, more precise operation, less postoperative pain, and shorter recovery time [28]. However, it has a steeper learning curve and is more complex than open pancreaticoduodenectomy (OPD) [29]. According to a study, LPD had a reduced operation time but an extended operative time in LPD compared to its open counterparts [29].

A study on lateral pancreaticojejunostomy for chronic pancreatitis found that the procedure provided pain relief, had a low morbidity rate, and no early postoperative deaths. However, long-term outcomes were poor based on the patient's health status, continued alcohol and narcotic use, employment status, subsequent hospitalizations for recurrent pancreatitis or its complications, subsequent operations required for complications of chronic pancreatitis, and postoperative deaths related to comorbid medical conditions or complications of chronic pancreatitis [30].

A study comparing open, laparoscopic, and robotic pancreatoduodenectomy found that LPD provided less trauma, less delayed gastric emptying, less transfusion, faster postoperative recovery, and shorter hospital stay compared to conventional OPD [31]. Both open and laparoscopic approaches to pancreaticojejunostomy have their advantages and disadvantages in terms of postoperative complications and long-term survival rates. It is essential to consider the patient's specific condition, the surgeon's experience, and the available resources when determining the most appropriate approach for the procedure.

Patient Selection

Patients with complications due to acute pancreatitis may be better suited for open procedures [32]. OPD may be preferred for patients with a higher risk of complications, such as those with a history of previous abdominal surgery or obesity [28]. LPD may be preferred for patients who require minimal access to surgery and have a lower risk of complications [28]. Laparoscopic surgery may offer comparable pain relief with additional benefits of minimal access surgery for carefully selected patients [33]. The choice between open and laparoscopic approaches to pancreaticojejunostomy depends on various factors, including the patient's specific condition, the surgeon's experience, and the available resources. Patients with a higher risk of complications may be better suited for open procedures. In comparison, those who require minimal access surgery and have a lower risk of complications may be better suited for laparoscopic procedures. It is essential to consult with a medical professional to determine the most appropriate approach for the procedure.

Recent advances

Robotics in Pancreatic Surgery

Overview of robotic-assisted techniques: In recent years, robotic-assisted surgery has emerged as a cutting-edge technology in pancreatic surgery, offering enhanced precision and dexterity to surgeons. Robotic systems, such as the da Vinci surgical system, enable the execution of complex procedures more efficiently than traditional laparoscopic approaches. In pancreaticojejunostomy, robotic-assisted techniques involve robotic arms controlled by the surgeon from a console, with high-definition 3D visualization providing an immersive view of the operative field [34]. The robotic approach to pancreaticojejunostomy follows a similar sequence to the laparoscopic technique. Trocars are placed to introduce robotic arms and a camera port into the abdominal cavity. The surgeon then manipulates the robotic instruments to perform the meticulous steps of the procedure, including identification and dissection of the pancreatic duct, selection of the jejunum for anastomosis, and the actual creation of the pancreaticojejunostomy. The robotic system's articulated instruments, which mimic the movements of the surgeon's hand with increased precision, facilitate delicate maneuvers and suturing [35].

Comparative analysis with open and laparoscopic approaches

Advantages of Robotic-Assisted Pancreaticojejunostomy

Enhanced dexterity: Robotic systems excel in providing enhanced dexterity through wristed instruments. These instruments offer a more excellent range of motion than traditional laparoscopic tools. This heightened dexterity is particularly advantageous in surgeries involving delicate procedures, such as pancreatic surgery. The ability to precisely manipulate tissues and perform intricate suturing is crucial in these scenarios. The robotic system's advanced design allows surgeons to navigate complex anatomical structures with increased flexibility and control, ultimately improving the overall surgical outcome [36].

3D visualization: One of the notable features of robotic surgical systems is their high-definition 3D visualization capability. This advanced visualization enhances a surgeon's depth perception during the procedure. The 3D view provides a more immersive and accurate representation of the surgical field. This improved spatial orientation is precious in tasks like anastomotic alignment, where precision is essential. The surgeon can perceive the depth and dimensions of structures more accurately, contributing to better decision-making and execution of surgical maneuvers [37].

Reduced learning curve: Despite the intricacies of robotic surgery, proponents argue that the learning curve associated with it might be less steep compared to traditional laparoscopy. This characteristic can be beneficial for surgeons transitioning from open surgical techniques. The intuitive interface and enhanced instrument control of robotic systems may allow surgeons to adapt more quickly to this technology. The reduced learning curve could make robotic surgery more accessible, enabling a broader range of surgeons to adopt and implement this advanced technology in their practices. This, in turn, may contribute to the broader integration of robotic-assisted procedures in various surgical specialties [38].

Considerations and Challenges

Cost: One significant barrier to the widespread adoption of robotic surgical systems is the substantial initial cost and ongoing maintenance expenses. Acquiring and maintaining robotic systems can be a significant investment for healthcare institutions. This includes the initial purchase of the robotic platform, ongoing maintenance, and the costs associated with specialized training for surgeons and staff. The financial implications may limit the accessibility of robotic-assisted surgery in specific healthcare settings, particularly those with budget constraints. Hospitals and healthcare providers must carefully weigh the potential benefits against the economic considerations when investing in robotic technology [39].

Operative time: Studies have indicated that robotic-assisted pancreatic surgery may be associated with longer operative times compared to traditional laparoscopic or open procedures. The increased time required for robotic surgeries can be attributed to factors such as the robotic system's setup, the robotic arms' docking, and the learning curve associated with mastering the technology. However, it's important to note that operative times can vary based on the surgeon's experience and proficiency with the robotic system. As surgeons become more adept at using the technology, operative times may decrease. Nevertheless, the longer duration of robotic surgeries is a factor that needs to be considered in the overall assessment of the feasibility and efficiency of these procedures [40].

Limited evidence on outcomes: While there is promise in the use of robotic surgery, more comprehensive and robust evidence is needed to compare outcomes with open and laparoscopic approaches, especially in specific procedures like pancreaticojejunostomy. The existing body of research may have limitations regarding sample size, study design, and standardization of outcomes. More high-quality, randomized controlled trials and long-term follow-up studies are essential to understand better the comparative effectiveness, safety, and long-term outcomes of robotic-assisted surgeries. This evidence is crucial for informing clinical practice guidelines and helping surgeons decide the most appropriate approach for specific surgical interventions [41].

Controversies and Debates

The current debates in the surgical community encompass a wide range of topics, including the adoption of robotic surgery, controversies in surgical oncology, vascular surgery, and the regionalization of emergency general surgery (EGS) care. The use of robotic surgery remains a topic of ongoing debate within the surgical community, as highlighted by a cross-sectional study involving Indian surgeons, revealing diverse opinions on its adoption and application [42]. In the realm of surgical oncology, the complexities associated with caring for cancer patients have ignited discussions on various facets of cancer treatment and surgical interventions, generating ongoing debates [43]. Within the broader field of general surgery, controversies extend to vascular topics such as prophylaxis against postoperative deep vein thrombosis and the impact of carotid surgery [44]. The proposal for the regionalization of EGS care, akin to trauma care, has been suggested to address concerns about access to care. However, this proposition has given rise to debates concerning its potential effects on the general surgery profession, conflicts between acute care surgeons and community surgeons, and the possibility of fragmented care [45]. Furthermore, ongoing debates on various aspects of general surgery practice are anticipated to shape the future landscape of the profession. These discussions underscore the dynamic nature of the surgical field, with professionals actively engaging in dialogues to navigate emerging challenges and advancements in surgical practice [46].

Future directions

Emerging Technologies and Techniques

Advanced imaging modalities: As technology progresses, there is a growing interest in leveraging advanced imaging modalities to enhance the precision and safety of pancreatic surgeries. Intraoperative imaging techniques, such as near-infrared fluorescence and contrast-enhanced imaging, offer real-time visualization of vascular structures and aid in identifying critical anatomical landmarks during procedures like pancreaticojejunostomy. Near-infrared fluorescence can enhance tissue contrast, allowing surgeons to navigate complex vascular structures better and minimize the risk of inadvertent damage. Conversely, contrast-enhanced imaging enhances the visibility of blood vessels and helps surgeons make more informed decisions during surgery. Integrating these advanced imaging modalities improves intraoperative decision-making and may lead to better outcomes for patients undergoing pancreatic surgery [47].

Artificial intelligence in surgical planning: The application of artificial intelligence (AI) in surgical planning represents a cutting-edge development in the field. AI algorithms can analyze preoperative imaging data, offering valuable insights to assist surgeons in personalized surgical planning. In the context of pancreatic surgery, AI can predict optimal suture placement, identify variations in anatomy, and anticipate potential challenges during pancreaticojejunostomy. By leveraging machine learning and data analysis, AI systems can provide surgeons with tailored recommendations, optimizing the surgical approach and potentially improving outcomes. The integration of AI in surgical planning is an exciting avenue that has the potential to enhance the efficiency and accuracy of pancreatic surgeries [48].

Nanotechnology for drug delivery: Nanotechnology has emerged as a promising avenue for drug delivery in the postoperative period following pancreatic surgery. Innovations in nanoscale drug carriers enable targeted and localized administration of therapeutic agents. This approach aims to enhance healing and reduce complications, such as pancreatic fistulas, which can be a significant concern after pancreatic surgery. Nanocarriers can be designed to release controlled therapeutic substances, improving treatment efficacy while minimizing systemic side effects. The application of nanotechnology in drug delivery represents a forward-looking strategy to address postoperative challenges and improve the overall recovery and well-being of patients undergoing pancreatic surgery [49].

Potential Advancements in Both Open and Laparoscopic Approaches

Open surgical approaches: The trajectory of surgical innovation is steering towards the refinement of minimally invasive open surgery techniques, focusing on reducing invasiveness even further. The future landscape may witness the deployment of smaller incisions, more sophisticated retractors, and improved instrumentation in open surgical procedures. This strategic evolution aims to mitigate tissue trauma and enhance patient recovery while retaining open surgery's familiarity and tactile advantages. By incorporating these refinements, surgeons seek to balance the benefits of minimally invasive approaches and the established efficacy of open procedures. This nuanced approach acknowledges the potential limitations of laparoscopic or robotic methods in some instances and aims to optimize patient outcomes by embracing the best aspects of both worlds [50].

Simultaneously, future developments in open surgery are poised to embrace enhanced intraoperative imaging technologies, ushering in a new era of precision and visualization. This may involve integrating cutting-edge tools, such as augmented reality, into the surgical field. Augmented reality can enhance intraoperative navigation by providing surgeons with real-time, 3D visualizations. Surgeons would have a comprehensive and dynamic view of the surgical site, allowing for more precise interventions. This advancement contributes to improved accuracy during procedures and opens avenues for enhanced training and collaboration among surgical teams. The integration of advanced imaging technologies in open surgery represents a promising frontier, poised to elevate the standards of surgical care and contribute to further advancements in patient outcomes and overall surgical efficiency [51].

Laparoscopic surgical approaches: The evolution of surgical techniques is poised for a paradigm shift with the increasing integration of robotic technologies into laparoscopic procedures. This convergence presents a promising synergy, combining the advantages of robotic systems such as enhanced dexterity and improved visualization with the minimally invasive nature of laparoscopy. Surgeons stand to benefit from heightened precision and control, navigating intricate anatomical structures with greater ease. This fusion of technologies may redefine the landscape of minimally invasive surgery, offering a more sophisticated and efficient approach to various medical interventions [52].

In parallel, strides in haptic feedback systems hold the potential to address a longstanding challenge in laparoscopic surgery - the lack of tactile feedback. Innovations in this domain aim to provide surgeons with a sense of touch, allowing them to feel the resistance and texture of tissues during minimally invasive procedures. This enhanced tactile information could significantly improve the surgeon's ability to make informed decisions and execute precise maneuvers, bridging the gap between traditional open surgery and the minimally invasive approach [53].

Moreover, telepresence surgery emerges as a transformative prospect as communication technologies advance. This entails surgeons remotely operating robotic systems for laparoscopic procedures, transcending geographical barriers, and potentially democratizing access to specialized surgical expertise. Telepresence surgery holds promise for expanding healthcare access and facilitates collaboration among experts, allowing real-time guidance and mentorship during complex procedures. This innovative approach to surgical care represents a leap forward in the global dissemination of surgical expertise, paving the way for a more interconnected and collaborative medical future [54].

## Conclusions

In conclusion, the surgical landscape of pancreaticojejunostomy is marked by a historical foundation in open surgery characterized by accrued experience and familiarity. However, this approach has drawbacks, notably increased morbidity and prolonged recovery, prompting a critical reassessment of alternative techniques. The advent of robotic-assisted surgery introduces a new dimension, offering enhanced dexterity and 3D visualization that can mitigate the challenges associated with traditional open and laparoscopic approaches. The reduced learning curve further positions robotic-assisted techniques as attractive, potentially broadening access to surgeons transitioning from open methodologies. Looking ahead, the future of pancreaticojejunostomy holds promise with emerging technologies such as advanced imaging and AI. These innovations aim to refine open and minimally invasive approaches, optimizing surgical outcomes and minimizing postoperative complications. Surgeons are poised to leverage technological advancements to tailor their approach based on patient characteristics and institutional resources in navigating this evolving landscape. The comprehensive understanding presented in this review serves as a guide for the surgical community, fostering informed decision-making and advancing the field of pancreatic surgery toward improved patient care and outcomes.
